# Specific brain activation patterns associated with two neuromuscular electrical stimulation protocols

**DOI:** 10.1038/s41598-017-03188-9

**Published:** 2017-06-02

**Authors:** Jennifer Wegrzyk, Jean-Philippe Ranjeva, Alexandre Fouré, Anne Kavounoudias, Christophe Vilmen, Jean-Pierre Mattei, Maxime Guye, Nicola A. Maffiuletti, Nicolas Place, David Bendahan, Julien Gondin

**Affiliations:** 1 Aix Marseille Univ, CNRS, CRMBM, UMR 7339, 13385 Marseille, France; 2 Aix Marseille Univ, CNRS, Laboratoire Neurosciences Intégratives et Adaptatives, UMR 7260, 13385 Marseille, France; 3 AP-HM, Hôpital de Sainte Marguerite, Service de Rhumatologie, Pôle Appareil Locomoteur, 13005 Marseille, France; 4 AP-HM, Hôpital de la Timone, CEMEREM, Pôle Imagerie Médicale, 13005 Marseille, France; 5 Schulthess Clinic, Human Performance Lab, 8008 Zurich, Switzerland; 6 University of Lausanne, Faculty of Biology and Medicine, Institute of Sport Sciences and Department of Physiology, Lausanne, Switzerland; 7 Institut NeuroMyoGène, Université Claude Bernard Lyon 1, INSERM U1217, CNRS UMR 5310 Villeurbanne, France

## Abstract

The influence of neuromuscular electrical stimulation (NMES) parameters on brain activation has been scarcely investigated. We aimed at comparing two frequently used NMES protocols - designed to vary in the extent of sensory input. Whole-brain functional magnetic resonance imaging was performed in sixteen healthy subjects during wide-pulse high-frequency (WPHF, 100 Hz–1 ms) and conventional (CONV, 25 Hz–0.05 ms) NMES applied over the *triceps surae*. Each protocol included 20 isometric contractions performed at 10% of maximal force. Voluntary plantar flexions (VOL) were performed as control trial. Mean force was not different among the three protocols, however, total current charge was higher for WPHF than for CONV. All protocols elicited significant activations of the sensorimotor network, cerebellum and thalamus. WPHF resulted in lower deactivation in the secondary somatosensory cortex and precuneus. Bilateral thalami and caudate nuclei were hyperactivated for CONV. The modulation of the NMES parameters resulted in differently activated/deactivated regions related to total current charge of the stimulation but not to mean force. By targeting different cerebral brain regions, the two NMES protocols might allow for individually-designed rehabilitation training in patients who can no longer execute voluntary movements.

## Introduction

Neuromuscular electrical stimulation (NMES) consists of a series of intermittent electrical stimuli applied over the muscle or the nerve trunk in order to elicit isometric muscle contractions. NMES has emerged as an efficient tool to induce activity-dependent plasticity in neural circuits in both healthy subjects^1, 2^ and hypoactive patients with stroke^3–5^. For instance, changes in corticospinal excitability resulting from a single session of NMES have been reported for a variety of upper^6, 7^ and lower^8^ limb muscles. Accordingly, functional magnetic resonance imaging (fMRI) investigations revealed a widespread brain activation pattern in response to NMES of different muscle groups, including the contralateral primary motor (M1) and sensory (S1) cortices, secondary somatosensory area (S2), supplementary motor area (SMA) and prefrontal cortex^9–13^.

Despite the promise of NMES as a tool for driving neuroplasticity and improving motor function^3–5^, the influence of stimulation parameters on the magnitude of sensory inputs to the brain has been scarcely investigated. A few studies have only reported a dose-response relationship between either stimulation intensity^10, 14^ or pulse frequency^15^ and brain activation patterns. From a neuromuscular point of view, NMES-induced isometric contractions may arise from the direct activation of motor axons (i.e., efferent pathway) and/or from the recruitment of motoneurons in the spinal cord through the depolarization of sensory axons (i.e., afferent pathway). It has been reported that NMES parameters, such as stimulation intensity, pulse frequency and pulse duration, affect the relative contribution of efferent and afferent pathways to force production^16^. Conventional (defined hereafter as CONV) NMES protocols typically consist of short pulses (<400 µs) applied at frequencies between 15 and 40 Hz and high stimulation intensities^17^. This combination of stimulation parameters primarily elicits contractions through the direct activation of motor axons, due to both the sensitivity of motor axons to short pulses and the antidromic collision at high stimulation intensities. Over the last decade, a new NMES protocol emerged in the literature as an alternative to CONV, consisting of wide pulses (~1 ms) delivered at frequencies higher than 80 Hz and at low stimulation intensities (evoking 5–10% of maximal voluntary contraction (MVC)) (defined hereafter as WPHF)^17–19^. Wide pulses favour the recruitment of afferent axons because they have a longer strength-duration time constant and a lower rheobase as compared to motor axons^20, 21^, whereas low stimulation intensities limit the antidromic collision in the activated motor axons. It has been suggested that WPHF could lead to the synaptic recruitment of motor units according to the size principle^18^, thereby potentially reducing muscle fatigue as compared with CONV. As a consequence, WPHF could be particularly advantageous for patients with central nervous system (CNS) damage who are highly fatigable and impeded to perform voluntary contractions. However, this potential advantage of WPHF over the CONV has not been consistently demonstrated^22–24^, probably due to inter-individual differences in the contribution of efferent pathways to motor unit recruitment^22^. Regardless of muscle fatigue, both the longer pulse duration and the higher stimulation frequency associated with WPHF led to a higher total current charge as compared to CONV^23, 24^. This might therefore enhance the sensory input to the CNS according to the dose-response relationship previously reported for both stimulation intensity^10, 14^ and pulse frequency^15^. Furthermore, a high inter-individual variability in force production has been recently reported for WPHF^23, 25^, which is likely related to the development of persistent inward currents in spinal motoneurons^16^ and the activity-dependent hyperpolarization of motor axons^22, 24^. Considering that the activity from receptors in muscles, tendons and skin may increase with force level^26–28^, brain activation patterns associated with WPHF might also be larger in subjects showing higher mean force^24, 25^.

In the present study, we aimed at investigating the cerebral activation pattern of both CONV and WPHF NMES protocols as compared to the activation pattern of voluntary (VOL) contractions matched for the same initial isometric force level. We hypothesized that both the total current charge and mean force would enhance the extent of sensory inputs to the brain during WPHF therefore resulting in higher brain activity than CONV.

## Results

Two subjects were excluded from the analysis due to either large head movements (>2.5 mm) or large susceptibility artefacts on the EPI images. Ultimately, data from 16 subjects were considered for analysis.

### Mean force and stimulation parameters

**Figure 1:**
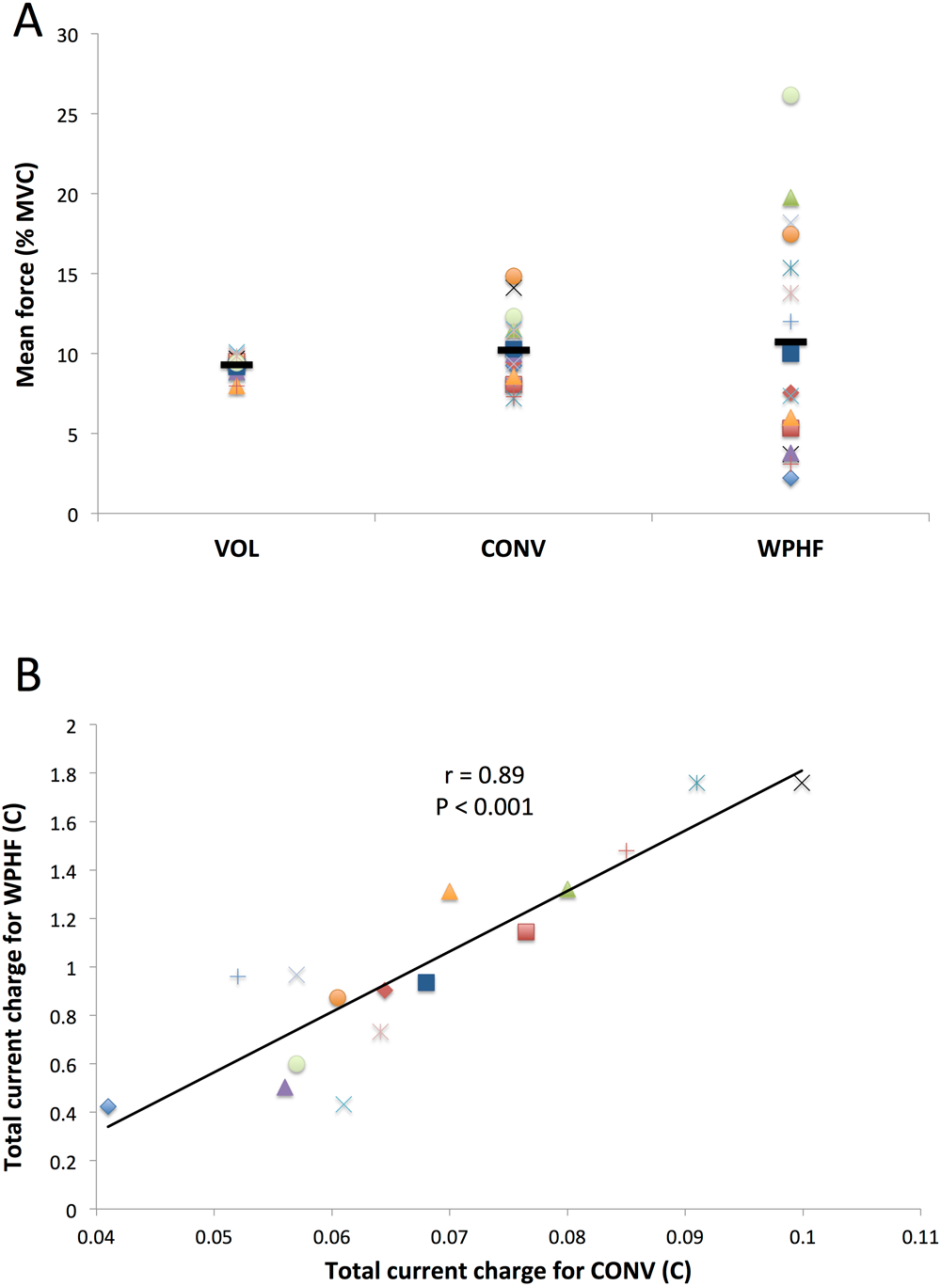
(**A**) Mean force recorded during VOL, CONV and WPHF protocols. The horizontal black bar represents the mean value for a given protocol whereas the symbols display individual values. Note the large inter-individual variability of mean force during WPHF. (**B**) Correlation between total current charge applied during WPHF and CONV protocols. Symbol colour and shape correspond to the same subject displays in panel A.

Mean force was not significantly different (*p* > 0.05) between the protocols (CONV: 10.2 ± 2.3% MVC; VOL: 9.3 ± 0.6% MVC; WPHF: 10.7 ± 7.1% MVC). As illustrated in Fig. 1A, mean force for WPHF showed a large inter-individual variability (CV = 66%). During WPHF, mean force was higher than 15% MVC for five subjects whereas four subjects displayed a mean force lower than 5% MVC. In contrast, mean force was only slightly variable among individuals during CONV (CV = 22%) and consistent during VOL (CV = 6%) protocols.

The stimulation intensity used to evoke 10% MVC was 6-fold higher (*p* < 0.05) for CONV (135 ± 31 mA) than for WPHF (25 ± 11 mA). Total current charge was 14-fold higher (*p* < 0.05) for WPHF (1.01 ± 0.43 C) as compared to CONV (0.07 ± 0.02 C) due to longer pulse duration and the higher stimulation frequency. Interestingly, total current charge of WPHF was positively correlated (r = 0.89, *p* < 0.001) with that of CONV (Fig. 1B). No significant correlation (*p* > 0.05) was found between mean force and total current charge for neither NMES protocol and between the mean forces of the two NMES protocols.

### Brain Activity

#### Activation patterns induced by voluntary and NMES protocols

**Figure 2:**
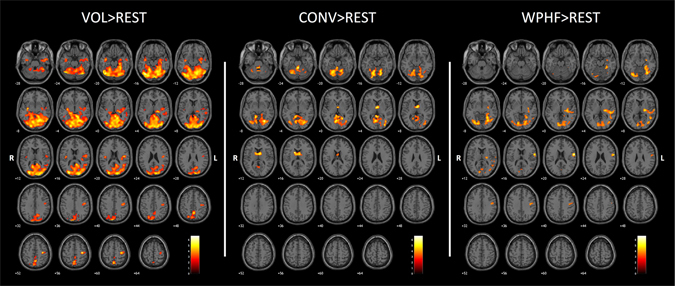
Group activation maps (*p* < 0.005; k = 20; FDR corrected at cluster level *p* < 0.05) during VOL, CONV and WPHF protocols, compared to rest, performed at 10% of maximal voluntary contraction force. Statistical maps were coregistered with the SPM-MNI single subject T_1_ images. The color scale represents the T values. R: right hemisphere; L: left hemisphere.

Figure 2 and Table 1 show significantly activated brain regions for each protocol as compared to rest.

**Table 1:** Brain regions activated during VOL, CONV and WPHF protocols compared to rest (*p* < 0.005; k = 20; FDR corrected at cluster level *p* < 0.05).

Protocols	Regions (Brodmann area)	Side	Cluster size (k)	MNI coordinates			T
				x	y	z	
VOL > REST	*Posterior cerebellum*	R	30064	38	−66	−18	7.75
	*Posterior cerebellum*	R		46	−62	−22	7.74
	*Precentral gyrus (4)*	L		−34	−18	54	7.31
	*Middle occipital gyrus (19)*	R		26	−84	14	7.09
	*Fusiform gyrus (19)*	L		−24	−64	−6	6.72
	*Anterior cerebellum*	L		−6	−46	0	6.62
	*Middle occipital gyrus (18)*	L		−22	−92	16	6.6
	*Fusiform gyrus (37)*	L		−50	−66	−12	6.54
	*Precuneus (7)*	L		−4	−56	50	6.48
	*Posterior cerebellum*	R		26	−78	−14	6.47
	*Lingual gyrus (17)*	R		18	−90	−4	6.43
	*Anterior cerebellum*	L		−18	−66	−10	6.41
	*Lingual gyrus (18)*	L		−22	−90	4	6.4
	*Lingual gyrus (19)*	L		−16	−54	−2	6.4
	*Orbital gyri (18)*	L		−38	−84	6	6.34
	*Lingual gyrus (18)*	R		8	−74	−6	6.32
CONV > REST	*Lingual gyrus (19)*	L	6364	−28	−68	−8	6.7
	*Posterior cerebellum*	R		22	−84	−16	6.37
	*Anterior cerebellum*	L		−2	−42	−24	5.98
	*Lingual gyrus (18)*	R		6	−68	2	5.95
	*Anterior cerebellum*	R		10	−48	−6	5.81
	*Cerebellum*	R		4	−44	4	5.76
	*Posterior cerebellum*	L		−18	−72	−16	5.73
	*Anterior cerebellum*	R		12	−56	−16	5.57
	*Anterior cerebellum*	L		−18	−56	−18	5.48
	*Lingual gyrus*	L		−24	−56	0	5.3
	*Posterior cerebellum*	R		20	−64	−18	5.22
	*Orbital gyri (18)*	L		−36	−86	4	5.08
	*Posterior cerebellum*	R		12	−72	−16	5.08
	*Anterior cerebellum*	L		−4	−48	2	4.9
	*Posterior cerebellum*	R		28	−68	−22	4.71
	*Lingual gyrus (19)*	R		36	−70	−6	4.68
	*Caudate nucleus*	R	798	14	6	16	6.28
	*Thalamus*	R		0	−6	6	6.13
	*Thalamus*	L		−6	−2	10	5.74
	*Thalamus*	R		8	−2	14	4.95
	*Caudate nucleus*	L		−18	−4	16	3.51
WPHF > REST	*Precentral Gyrus (4)*	L	4988	−62	−2	20	6.12
	*Fusiform gyrus (19)*	L		−32	−64	−10	5.23
	*Fusiform gyrus (19)*	L		−28	−66	−8	4.88
	*Posterior cerebellum*	R		32	−70	−10	4.77
	*Putamen*	L		−24	−18	8	4.77
	*Parahippocampal gyrus (19)*	L		−26	−50	−6	4.75
	*Lingual gyrus (18)*	R		16	−78	−6	4.71
	*Anterior cerebellum*	L		−24	−44	−14	4.68
	*Fusiform gyrus (37)*	L		−28	−40	−16	4.65
	*Lateral globus pallidus*	L		−28	−20	0	4.53
	*Middle temporal gyrus (22)*	L		−46	−30	4	4.45
	*Anterior cerebellum*	L		−32	−52	−12	4.27
	*Putamen*	L		−32	−14	−2	4.27
	*Lingual gyrus (18)*	R		10	−86	0	4.25
	*Parahippocampal gyrus (36)*	L		−30	−36	−14	4.24
	*Lingual gyrus (18)*	L		−6	−86	8	4.23

VOL induced significant activation within the contralateral M1 (BA4), S1 (BA3), S2 (BA43), putamen, thalamus, lateral globus pallidus and posterior cingulate gyrus (BA 31). Moreover, bilateral activations were observed in the primary visual cortex (BA 17), lingual gyrus (BA 18,19), fusiform gyrus (BA 37), cerebellum, precuneus (BA 7), posterior cingulate gyrus (BA 30, 31), insula (BA 13), hippocampus, posterior cingulate area (BA23) and ipsilateral amygdala. No region was found to be deactivated during VOL relative to the resting period (data not shown).

CONV resulted in significant bilateral activations within the caudate nuclei, thalamus, cerebellum, lingual gyrus (BA 18, 19) and anterior cingulate gyrus (BA 30). Several areas were shown to be deactivated during the CONV as compared to the resting period, especially the superior and inferior frontal gyri, the bilateral supramarginal gyrus (BA 40), the inferior parietal lobule (BA 7, 40), the angular gyrus, the contralateral cingulate gyrus (BA 24) and the precuneus (Supplemental Fig. 1).

WPHF resulted in significant activation in contralateral M1, S1, S2, premotor cortex (BA 6), putamen, thalamus, lateral globus pallidus, insula (BA 13), hippocampus, temporal gyrus (BA 22), amygdala and in the ipsilateral cerebellum. Bilateral activation was further observed in the lingual gyrus (BA 18 and 19), primary visual cortex (BA 17) and fusiform gyrus (BA 37). The contralateral superior frontal gyrus, the postcentral gyrus and the bilateral caudate nuclei were found to be deactivated during WPHF relative to the resting condition (Supplemental Fig. 1).

**Figure 3:**
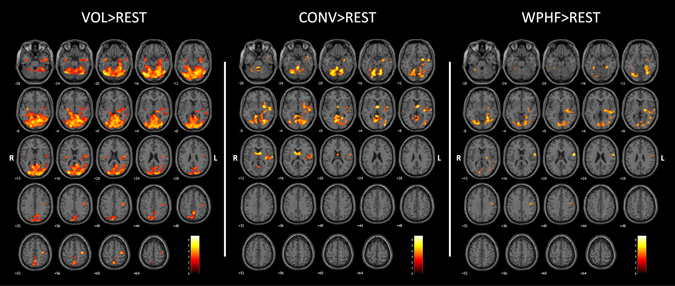
Group activation maps (*p* < 0.005; k = 20; FDR corrected at cluster level *p* < 0.05) during VOL, CONV and WPHF protocols compared to rest, when considering the respective mean force as a regressor. Statistical maps were coregistered with the SPM-MNI single subject T_1_ images. The color scale represents the T values. R: right hemisphere; L: left hemisphere.

Mean force regression resulted in almost identical activation patterns for VOL (Supplemental Table 1 and Fig. 3) and WPHF (Supplemental Table 1 and Fig. 3). For CONV, an additional cluster (k = 2126) reached significance within the contralateral M1, S1, S2, putamen, insula, temporal gyrus (BA 22, 38), globus pallidus, amygdala and transverse temporal gyrus (BA 41). Total current charge regression (Fig. 1B) resulted in similar brain activation patterns for CONV and WPHF (data not shown).

#### Direct comparison of activation patterns between protocols

**Figure 4:**
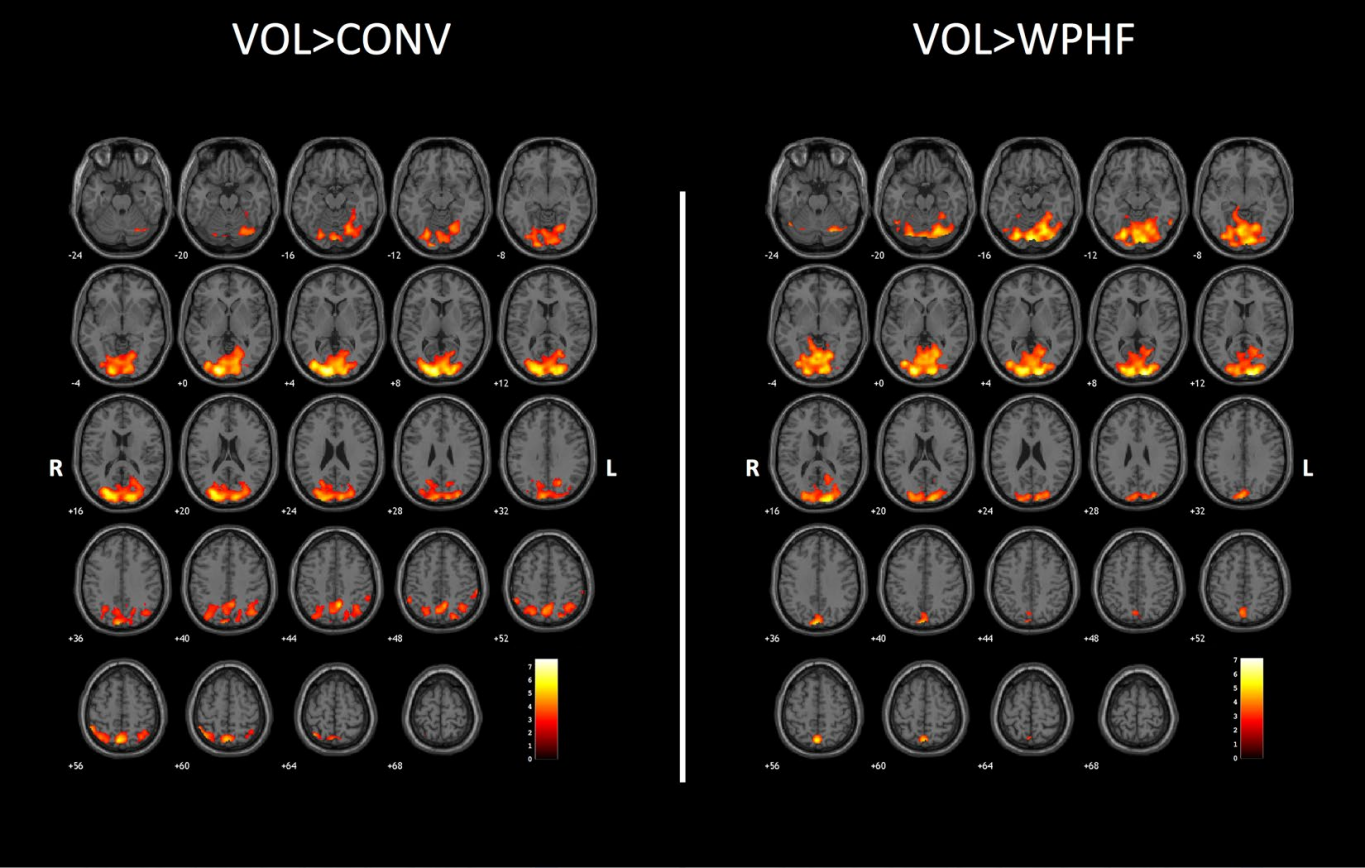
Whole brain contrast maps (*p* < 0.005; k = 20; FDR corrected at cluster level *p* < 0.05) between VOL and the two NMES protocols. Statistical maps were coregistered with the SPM-MNI single subject T_1_ images. The color scale represents the T values. R: right hemisphere; L: left hemisphere.

**Figure 5:**
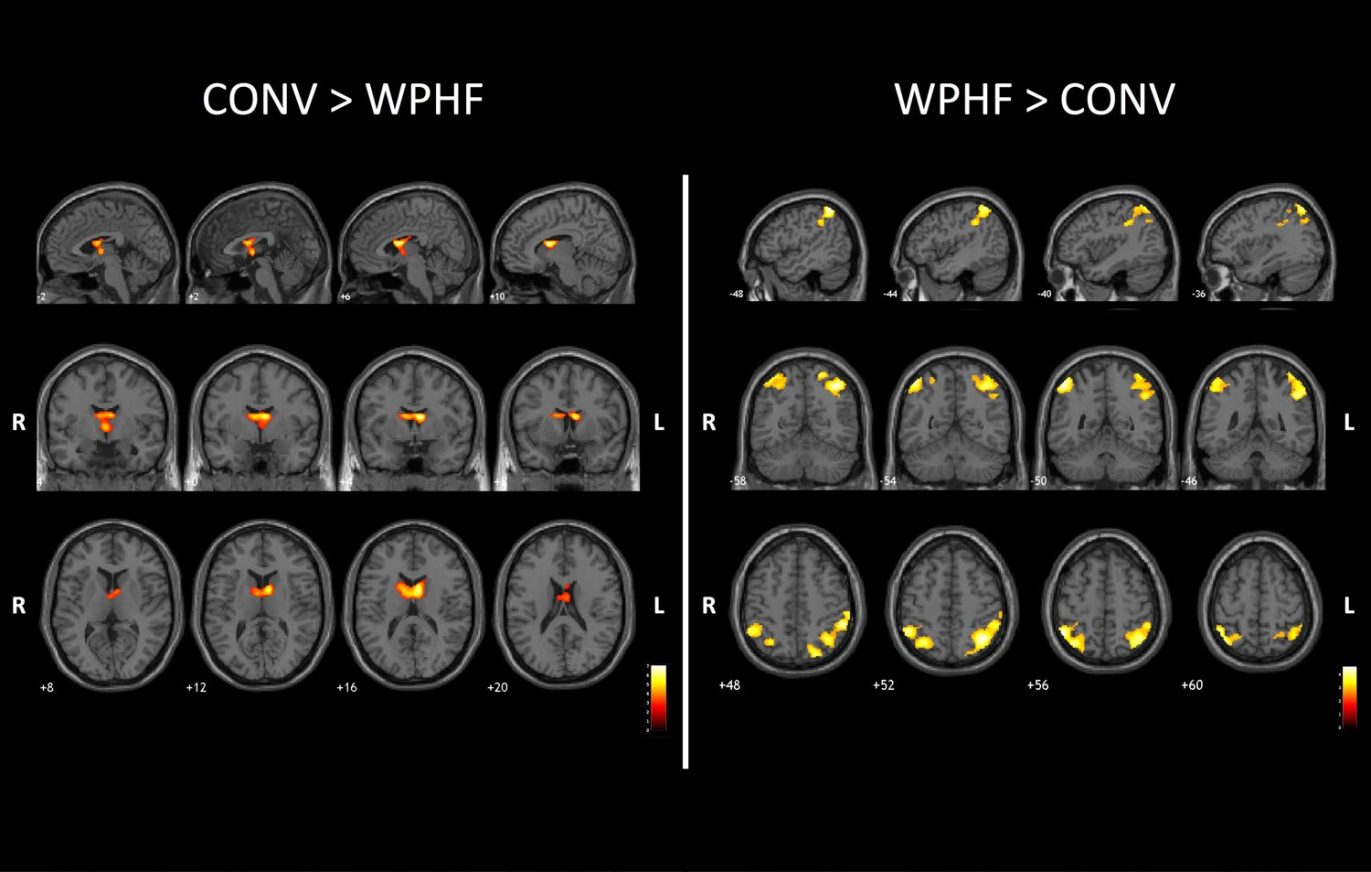
Whole brain contrast maps (*p* < 0.005; k = 20; FDR corrected at cluster level *p* < 0.05) between the two NMES protocols (top, sagittal view; middle, coronal view; bottom, axial view). Statistical maps were coregistered with the SPM-MNI single subject T_1_ images. The color scale represents the T values. R: right hemisphere; L: left hemisphere.

Brain activation contrast maps for all three protocols are displayed in Figs 4 and 5 and Table 2. As compared to NMES, VOL showed larger activations within the cerebellum, bilateral precuneus (BA7), cuneus (BA 17), lingual gyrus (BA 18) and middle occipital gyrus (BA 18, 19) (Fig. 4 and Supplemental Table 2).

**Table 2:** Differences in brain activation between the two NMES protocols (*p* < 0.005; k = 20; FDR corrected at cluster level *p* < 0.05).

Contrasts	Regions (Brodmann area)	Side	Cluster size (k)	MNI coordinates			T
				x	y	z	
CONV > WPHF	*Caudate nucleus*	L	723	−8	4	14	7.04
	*Thalamus*	R		0	−6	2	5.28
	*Caudate nucleus*	R		4	0	14	5
	*Caudate nucleus*	R		10	4	16	4.92
WPHF > CONV	*Superior parietal lobule (7)*	L	1679	−40	−58	52	4.57
	*Superior parietal lobule (7)*	L		−20	−62	68	4.54
	*Inferior parietal lobule (40)*	L		−54	−42	48	4.49
	*Supramarginal gyrus (40)*	L		−48	−48	40	4.17
	*Precuneus (7)*	L		−22	−78	50	4.04
	*Inferior parietal lobule (40)*	L		−62	−30	48	3.91
	*Superior parietal lobule (7)*	L		−44	−48	64	3.91
	*Inferior parietal lobule (40)*	L		−50	−44	56	3.75
	*Angular gyrus (39)*	L		−48	−70	40	3.22
	*Postcentral gyrus*	L		−42	−40	68	3.12
	*Inferior parietal lobule (40)*	L		−34	−38	34	3.07
	*Inferior parietal lobule (40)*	L		−36	−38	42	3.04
	*Superior occipital gyrus (19)*	L		−32	−72	40	2.97
	*Cuneus (19)*	L		−20	−80	40	2.96
	*Inferior parietal lobule (40)*	L		−34	−40	38	2.92
	*Angular gyrus (39)*	L		−42	−58	40	2.91
	*Inferior parietal lobule (40)*	R	1194	48	−52	56	4.52
	*Precuneus (7)*	R		34	−64	56	4.2
	*Superior parietal lobule (7)*	R		40	−60	60	4.15
	*Inferior parietal lobule (40)*	R		44	−40	38	3.59
	*Inferior parietal lobule (40)*	R		48	−38	38	3.5
	*Postcentral gyrus*	R		28	−26	40	3.27
	*Cingulate gyrus (31)*	R		20	−32	38	3.25
	*Superior occipital gyrus (19)*	R		36	−70	40	3.15
	*Angular gyrus (39)*	R		38	−66	40	3.13
	*Superior parietal lobule (7)*	R		20	−64	66	3.03
	*Inferior parietal lobule (40)*	R		34	−30	36	2.99
	*Inferior parietal lobule (7)*	R		32	−44	54	2.98
	*Postcentral gyrus*	R		26	−22	40	2.89
	*Superior parietal lobule (7)*	R		20	−56	60	2.88
	*Inferior parietal lobule (7)*	R		32	−40	56	2.84

CONV showed larger bilateral activations within the thalamus and caudate nuclei as compared to both WPHF and VOL (Fig. 5, Table 2 and supplemental Table 2). In addition, CONV resulted in greater activation in the ipsilateral putamen as compared to VOL (supplemental Table 2).

WPHF showed less deactivation than CONV within the bilateral precuneus (BA 7), supramarginal gyrus (BA 40), angular gyrus (BA 39), inferior and superior parietal lobules (BA 7, 40), contralateral cuneus (BA 19) and ipsilateral cingulate gyrus (BA 31) (Fig. 5 and Table 2). No additional cluster was found for WPHF as compared to VOL.

Mean force regression resulted in almost identical activation patterns between the protocols (data not shown). In contrast, brain activation between the NMES protocols was no longer different after total current charge regression.

## Discussion

In the present whole-brain fMRI study, we aimed at comparing brain activation patterns elicited by two NMES protocols and one voluntary isometric protocol. The modulation of the NMES parameters resulted in differently activated/deactivated regions related to total current charge of the stimulation but not to mean force produced during the protocols.

The voluntary protocol resulted in an increased activity of controlateral primary and sensory motor cortices as previously described for plantar flexions^29–32^. In addition, we observed a bilateral activation in the visual cortex and cerebellum associated with an increased activity in contralateral subcortical regions - potentially related to the experimental design that required active visuomotor coordination^26, 33, 34^. Contrary to previous functional brain investigations performed on lower limbs^12, 29, 31, 35^, the SMA was presently not activated probably due to exercise type related differences in proprioception (isometric *vs*. dynamic contraction). Our study design involved sustained isometric contractions for which muscle length did not change, thereby resulting in less fusimotor drive^36, 37^ as compared to dynamic actions^12, 29, 35^. In accordance with our results, Keisker and colleagues^38^ only detected SMA activity during intermittent contractions but not during sustained isometric contractions at an equivalent force level. Therefore, SMA activity seems to play a key role when a motor task involves rapid transitions between muscle activation and recovery periods^36, 37^.

The two NMES protocols resulted in an increased activity of the contralateral M1 and S1, a finding consistent with previous NMES investigations^9–13, 39^. The activation of M1 in the absence of voluntary neural drive has been related to inputs from the S1 and thalamus^40^ through the activation of proprioceptors and mechanoreceptors *via* ascending sensory pathways^10–12^. Tactile stimulation applied on the glabrous hand skin^41^ as well as a tendinous vibration - known to specifically activate muscle spindle endings - have previously elicited contralateral S1 and M1 activation. In contrast to previous studies showing a bilateral S2 activation^11–14, 42–45^ we only observed a contralateral S2 activation in response to NMES. The discrepancies could be explained by the fact that we delivered the electrical stimuli over the muscle belly using large electrodes and not over the nerve trunk using small electrodes. Instead of depolarizing relatively bundled afferent fibers in a time-locked manner, the heterogeneous spatial distribution of afferents during muscle stimulation likely resulted in less synchronous neuronal inputs to the brain^16^. Therefore, the temporal dispersion and the composition of the afferent volley might have minimized the BOLD responses in the ipsilateral S2 through decreased inputs from the contralateral S2 via transcallosal fibers^46^ or by direct thalamocortical projections^47, 48^.

Accordingly, we and others^39^ only observed a contralateral activation in the insula after NMES whereas bilateral activation has been largely reported for peripheral nerve NMES^12, 43–45^. These findings suggest that the activity in the ipsilateral insula might be related to the ipsilateral activation of S2^12^. Finally, a reduced processing of somatosensory information when NMES is applied over the muscle belly might also explain the absence of SMA and anterior cingulate activity in our study as compared to NMES of median and tibial nerves during painful and non-painful stimulation^43^. In addition, the use of electrically-evoked isometric contractions in the current study prevented ankle joint movements, known to induce an increased activity within the SMA^12, 29, 35^. Overall, it is very likely that the site of NMES application influences the brain activation patterns. However, several studies showed that NMES applied via nerve stimulation generates more discomfort than NMES *via* muscle stimulation^16, 49^, which results in higher activation of pain-sensitive brain regions (e.g., anterior cingulate cortex)^50, 51^. Given that discomfort is one of the main limitations of NMES^52^, future studies should investigate whether NMES applied over a nerve trunk would be a suitable strategy for maximizing brain activation.

When contrasting both NMES protocols, we found that relative to CONV, WPHF stimulation elicited less deactivation within regions of the default mode network^53^, highlighted by the negative contrasts (i.e., REST > CONV and REST > WPHF) such as precuneus, supramarginal gyrus, angular gyrus, inferior and superior parietal lobules. All those regions have been associated with illusory movement sensations preferentially activated by muscle spindle endings through mechanical vibration of the muscle tendon^54, 55^. We demonstrated that the bilateral S2 was less deactivated during WPHF than during CONV likely as a result of an increased total current charge and sensory processing. Our findings therefore support previous studies showing that bilateral S2 is involved in high-order functions such as sensorimotor integration and attention^14, 44, 56, 57^. We also found that the precuneus was less deactivated during WPHF as compared to CONV. It has been reported that the precuneus receives tactile and proprioceptive inputs^58^ and may act as a processor of proprioception stimuli^14, 59^. Interestingly, when considering total current charge as a regressor, activation in both S2 and precuneus was no longer different between the two NMES protocols suggesting that the different activation/deactivation profiles are related to the extent of sensory inputs to the brain.

In the present study, the WPHF-evoked force was highly variable among subjects (Fig. 1A), despite the careful setting of an initial target force of 10% MVC. This finding is consistent with our previous investigations^23–25, 60^ and has been related to the development of persistent inward currents in spinal motoneurons^16^ and to the activity-dependent hyperpolarization of motor axons^22, 24^. In order to take into account the force differences among protocols and subjects, the general linear model analysis was performed with an additional parametric regressor in the design matrix modeling the individual mean force (in % MVC, averaged over 20 seconds) for each subject, each contraction and each condition. However, such “mean force” regression did not eliminate the differences in brain activation patterns between the two NMES protocols, thereby indicating that the level of evoked force did not influence the brain activation patterns associated with each protocol. Compared to previous studies showing a dose-response relationship between isometric force and brain activity^26–28^, we limited the potential effect of the mean force on brain activation by investigating only force levels <25% MVC, given the large head movements artefacts when plantar flexion force was above 30% MVC. Accordingly, Van Duinen *et al*.^26^ found no significant increase in brain activity when finger abduction force increased from ∼9 to ∼17% MVC. Experimental setups in which knee joint flexion is at around 45°^31^ would minimize WPHF-related head movements and allow for the investigation of WPHF-induced brain activity over a wider range of forces.

The specific brain activation pattern associated with CONV consisted in a higher bilateral activation in thalamus and caudate nuclei as compared to WPHF. Painful lower limb heat stimulation has previously resulted in such activation^61^, however, neither NMES protocol was considered as painful. Interestingly, bilateral responses within the basal ganglia have been reported in response to tactile stimulation and/or tendon vibration^54, 58, 62^, indicating a subcortical processing of afferent stimuli. Given the considerably lower total current charge for CONV than for WPHF, the hyperactivation in these regions might reflect a better discrimination of the electrical stimuli due to a reduced number of sensory inputs and/or an increased time delay between the repetitive stimulation pulses (i.e., 40 ms *vs*. 10 ms for CONV and WPHF, respectively). Interestingly, CONV also resulted in greater activation in caudate nuclei, thalami and putamen than VOL, thereby highlighting the specificity of this electrically-evoked neuronal network.

This proof-of-concept study might open new perspectives for driving neuroplasticity and improving motor function in patients with brain diseases. Indeed, both NMES protocols elicited brain activation within a wide network of cortical and subcortical structures close to that activated during repeated isometric voluntary contractions. It has been previously shown that increased sensory input can improve motor function and learning by increasing the excitability of the neuronal path projecting to muscles and joints wherein the sensory receptors are activated^63^. One could therefore speculate that the two NMES protocols might induce a specific brain plasticity due to the differences in the magnitude of sensory inputs to the brain. On that basis, the selective activation of afferent pathways could be viewed as an attractive way to promote neuroplasticity in specific brain areas by simply modulating the stimulation parameters. For example, the specific activation of basal ganglia during CONV might be relevant for minimizing proprioceptive deficits in elderly individuals^62^ or in patients suffering from movement disorders such as Parkinson’s and Huntington’s disease^64, 65^. However, our results cannot yet be translated into clinical applications and personalized rehabilitation since only young and healthy individuals were tested. Indeed, brain activation patterns associated with either voluntary or electrically-evoked contractions may differ when comparing healthy individuals with patients with CNS damage^47, 66–68^. Furthermore, the present findings cannot be used to infer that one of the two NMES protocols is more efficient in terms of functional recovery because we only investigated the influence of a single NMES session on brain activation. It is noteworthy that our study was not designed to address whether chronic NMES application would be able to re-direct and strengthen brain connectivity in patients who lost movement control following CNS damage. Further investigations are clearly needed to determine whether and to which extent NMES-induced neuroplasticity could be related to the applied stimulation parameters. It remains also to be demonstrated whether these two NMES protocols performed at relatively low force levels (i.e., 10% MVC) would be suitable for minimizing the alterations in cardiovascular fitness and the reductions in both muscle mass and bone mineral density usually observed in patients with CNS damage^69^ or should be better combined with other NMES and/or exercise modalities. Finally, considering that muscle fatigue remains one of the main limitations of NMES in clinical settings^52^, it is still unclear whether WPHF would allow to generate more fatigue-resistant contractions as compared with CONV.

In conclusion, we demonstrated that both NMES-induced isometric contractions resulted in widespread brain activation patterns including sensorimotor areas and subcortical structures in accordance with the activation pattern of voluntary movements. We also showed specific brain activation and deactivation for each NMES protocol that could be related to the total current charge applied over the muscle belly and thereby to the magnitude of the sensory volley. On the contrary, mean force did not account for the differential brain activation between the two NMES protocols. Our results might encourage the development of individually-designed stimulation protocols in the future to target specific/impaired cerebral brain regions.

## Methods

Eighteen healthy subjects (12 men, 6 women; age: 26 ± 5 years; height: 173 ± 9 cm; weight: 66 ± 8 kg) without neurological injury or disease, gave written informed consent to participate in this study. Subjects were not inscribed in any exercise program and instructed to refrain from intense and non-familiar physical activities for 48 h before the experiment. All experimental procedures were approved by the Local Human Research Ethics Committee Sud Méditerranée I (n° 2012-A01265-38) and in conformity with the Declaration of Helsinki. Informed consent has been also obtained for publication of identifying images.

### Study design

The experiment consisted of two sessions, separated by at least seven days. The first session, performed outside the scanner, was used to familiarize the subjects with the protocols. Maximal voluntary isometric plantar flexion force was determined after a 5-min warm-up period including submaximal voluntary contractions (gradually increasing from 10% to 75% of MVC). The individual stimulation intensity was subsequently determined according to 10% of the MVC for each NMES protocol (see below). The corresponding stimulation intensities were maintained during 5 electrically-evoked contractions lasting 20 s to ensure toleration of the stimulation. In addition, subjects were asked to perform 5 voluntary isometric contractions of 20 s at 10% MVC.

In the second session, subjects were lying in a supine position on the MR scanner bed. This session lasted ~2 h and included i) a warm-up consisting of 5–7 submaximal plantar flexion contractions of 5 s, ii) the assessment of isometric MVC force, iii) the adjustment of NMES intensity by using 2-s testing trains and iv) the three protocols (i.e., CONV, VOL, WPHF) performed in a randomized order^24^.

### Experimental procedure

#### Force recordings

**Figure 6:**
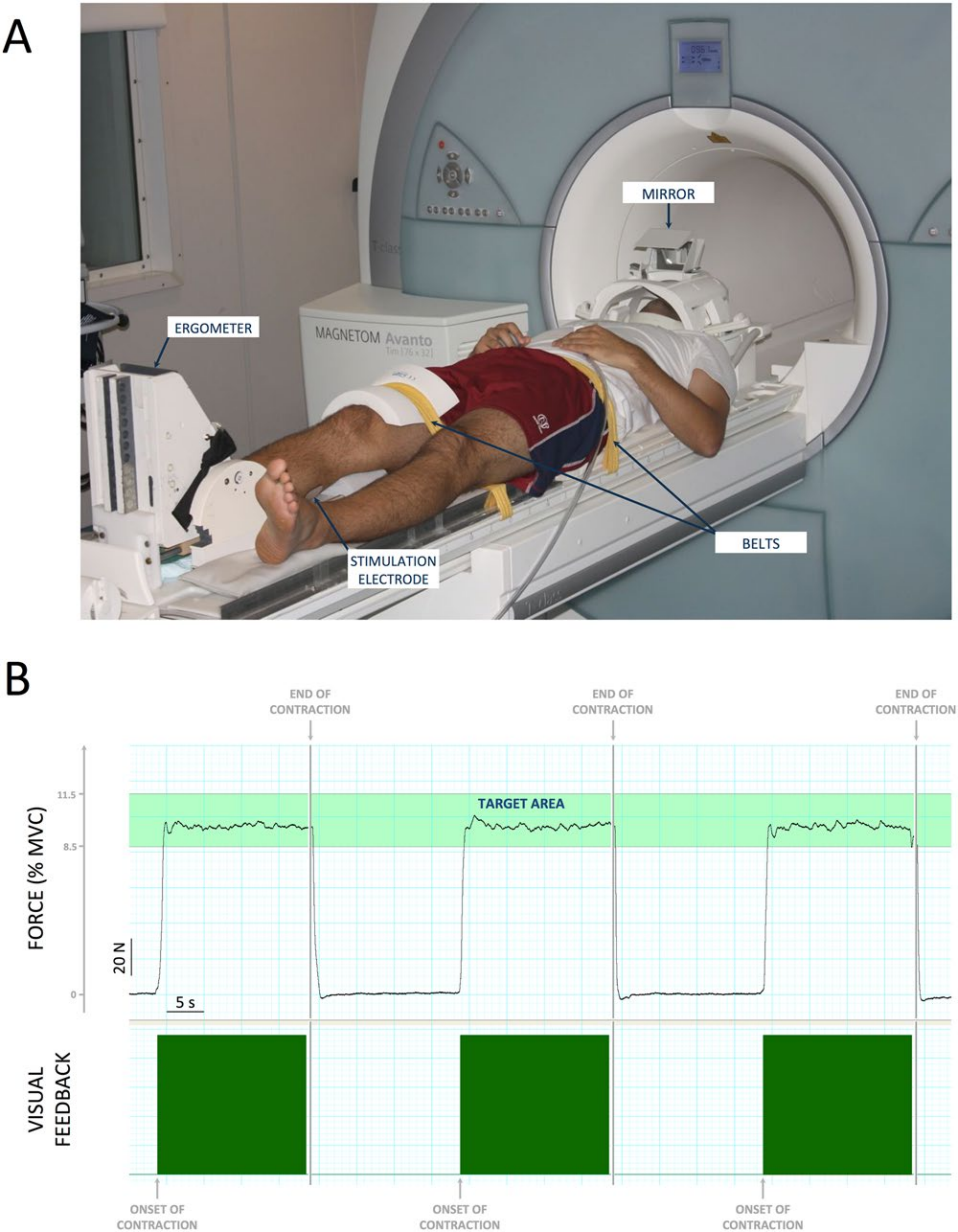
(**A**) Experimental setup: Custom-made ergometer to record force production during the three protocols within a whole body 1.5 T MR scanner. (**B**) Visual feedback used during the voluntary protocol (to reach the 10% MVC target force level) including typical force recordings for three submaximal voluntary contractions.

Voluntary and electrically-evoked plantar flexion forces were recorded using a custom-made MR compatible ergometer consisting of a foot pedal coupled with a force transducer and amplifier^24^. All experiments were performed on the right *triceps surae* while subjects lay supine on the MR scanner bed. The right knee of the subject was fixed at 170° (180° = full extension) and the forefoot and heel were firmly strapped to the pedal. The foot was securely held in position with an ankle angle of 90° while the knee and the hip were securely fixed with nonelastic belts to the bed in order to limit force generation by other muscle groups than the *triceps surae* (Fig. 6A) and head movements during MRI acquisitions.

Before entering the MR scanner, subjects were instructed to perform two 5-s isometric VOL plantar flexion MVCs separated by a 120-s resting period in between. If variations in MVC exceeded 5%, further trials were performed. The force signal was acquired on a personal computer at a sampling frequency of 2 kHz using the Powerlab 16/36 data acquisition system (LabChart 7, ADInstruments, Sydney, Australia).

#### NMES

Two flexible surface electrodes of 5 × 13 cm and 5 × 9 cm (STIMEX schwa-medico GmbH, Ehringshausen, Germany) were placed on the right *triceps surae*. The proximal and largest electrode was placed over the *gastrocnemii* at approximately the point of the largest circumference. The distal electrode was placed over the soleus muscle below the bottom of the medial gastrocnemius muscle belly^24^. Monophasic rectangular electrical stimuli were delivered using a constant-current stimulator (Digitimer DS7A, Hertfordshire, UK; maximal voltage: 400 V). Pulse frequency and duration were 100 Hz and 1 ms for WPHF *vs*. 25 Hz and 0.05 ms for CONV. At the beginning of each protocol, 2-s testing trains with stimulation characteristics corresponding to the respective NMES protocols (see above) were applied by gradually adjusting the stimulation intensity until 10% of the MVC force were reached (tolerance range: 8.5–11.5% MVC). Once determined, the stimulation intensity was kept constant throughout the protocol so that fluctuations in force output during the stimulation affected individual mean force. Individual stimulation intensities were consistently recorded for both protocols.

#### Protocols

Subjects were instructed to lie relaxed with their hand resting upon the abdomen and to keep their eyes open during each protocol. As previously described^24^, each protocol consisted of a block design including 20 isometric plantar flexion contractions (duration: 20 s; intensity: 10% MVC) separated by rest periods of 20 s. For both NMES protocols, stimulation trains were triggered by the Powerlab system interfaced to the MR scanner. Subjects were explicitely asked to remain completely relaxed during NMES. Both NMES protocols were not considered painful for the subjects according to subjective perceptions. For the VOL protocol, real-time visual feedback of force production was projected on a screen that was perceived by the subject *via* a mirror mounted on the head coil (Fig. 6A). A light green coloured area with the lower and upper limit corresponding to 8.5% and 11.5% MVC indicated the target force level. In addition, a dark green box indicated the onset and contraction duration while a vertical grey line further marked out the end of the contraction. A continuous black line displayed the actual force (Fig. 6B).

### fMRI data acquisition

Experiments were performed in a 1.5 T whole-body MR scanner (Siemens Avanto MR system; Siemens AG, Erlangen, Germany) equipped with a 12-channel head coil (Fig. 6A). The subjects’ heads were fixed with foam in order to limit head motion. Functional images were acquired with the following parameters: single-shot gradient-echo EPI sequence; 30 contiguous axial slices; slice thickness = 4 mm; TR = 3.3 s; TE = 60 ms; flip angle = 90°; FOV = 256 × 256 mm^2^; matrix = 64 × 64 (i.e., nomimal voxel size = 4 × 4 × 4 mm^3^). Four dummy scans were performed at the beginning of each functional measurement in order to achieve magnetization steady state. For each protocol, a total of 240 brain volumes (i.e., 120 at rest and 120 during contractions) were acquired in a single run lasting ∼13 min. The MR acquisition was synchronized to the stimulation procedure using the Powerlab system. All fMRI experiments were performed on the same day with identical positioning of the stimulation electrodes and the MRI coil. The three protocols were separated by a 15-min recovery period in order to minimize potential fatigue effects^24^. At the end of the three protocols, 3D T_1_-weighted anatomical images were obtained.

### Data and statistical analysis

#### Force & stimulation parameters

The highest peak force value achieved across the different trials was considered as the MVC. For each protocol, mean force was calculated over a 20-s window and was then averaged across the 20 blocks. The corresponding value was expressed relative to the individual MVC value (% MVC). The coefficient of variation (CV) was calculated for each protocol in order to assess inter-individual variability of mean force^25^. The total current charge representing the total amount of current delivered during each NMES protocol was calculated as follows:

Total current charge (C) = intensity (A) × pulse duration (s) × number of pulses

Considering the different stimulation frequencies between the two NMES protocols, the total number of pulses was 40 000 and 10 000 for WPHF and CONV, respectively.

A one-way repeated measures ANOVA was performed to assess differences in mean force among the three protocols. Paired t-test was used to compare the total current charge between the two NMES protocols. Correlations between selected variables were tested with Pearson coefficient. The level of significance was set at *p* < 0.05.

#### fMRI data

Analysis was performed using Statistical Parametric Mapping (SPM12; Welcome Department of Imaging Neuroscience, London, UK) implemented in Matlab (Matlab R2014a). The initial images were pre-processed (slice timing, realignment) before spatial normalization to the Montreal Neurological Institute (MNI) using the conventional SPM procedure, leading to an interpolated fMRI dataset with voxel size of 2 × 2 × 2 mm^3^. Then spatial filtering was applied (Gaussian filter with 10 mm FWHM) before performing GLM statistics.

First level analysis used a general linear model with a conventional boxcar design modelling the 20 blocks of 20-s resting period followed by 20-s of contraction convolved with a canonical haemodynamic response function. The six motion parameters (three rotations and three translations) were included in the design matrix as regressors in order to account for each subject’s head movement. The mean translational and rotational displacements were −0.16 ± 0.71 mm and 0.002 ± 0.009°; −0.11 ± 0.51 mm and 0.002 ± 0.009° and −0.06 ± 0.51 mm and 0.002 ± 0.008° for VOL, CONV and WPHF, respectively. Six contrasts (VOL > REST; CONV > REST, WPHF > REST, REST > VOL, REST > CONV and REST > WPHF) were then generated for each subject.

Second level analyses used each individual contrast images to obtain group activation patterns for each protocol (one-sample t-test, *p* < 0.005; k = 20; FDR corrected at cluster level *p* < 0.05). Two separate regression analyses were performed to test if differences in brain activity could be explained by 1) total current charge (in coulomb, C) or 2) individual mean force (in % MVC) both of which varied among protocols and between subjects^23–25^.

In addition, a one-way ANOVA – within subject (*p* < 0.005; k = 20; FDR corrected at cluster level *p* < 0.05) was performed to highlight differences in brain activity between protocols. Mean force and total current charge were taken into account as two independent regressors.

Location of the activation clusters was determined using the atlas of Talairach and Tournoux^70^ as well as the AAL atlas^71^ in order to provide both functional and anatomical labeling.

## Electronic supplementary material

SUPPLEMENTAL DATA
