# Supplementary material for: Specific brain activation patterns associated with two neuromuscular electrical stimulation protocols
Source: Sci Rep. 2017 Jun 2;7:2742. doi: 10.1038/s41598-017-03188-9 (PMC5457446; doi:10.1038/s41598-017-03188-9)
Supplement: Supplementary file 1 — SUPPLEMENTAL DATA [file 41598_2017_3188_MOESM1_ESM.pdf]

Specific brain activation patterns associated with two neuromuscular electrical stimulation protocols

Jennifer Wegrzyk[1], Jean-Philippe Ranjeva[1], Alexandre Fouré[1], Anne Kavounoudias[2], Christophe Vilmen[1], Jean-Pierre Mattei[1,3], Maxime Guye[1,4], Nicola A. Maffiuletti[5], Nicolas Place[6], David Bendahan[1], Julien Gondin[1,7,*]

[1] Aix Marseille Univ, CNRS, CRMBM, UMR 7339, 13385, Marseille, France.

[2] Aix Marseille Univ, CNRS, Laboratoire Neurosciences Intégratives et Adaptatives, UMR 7260, 13385, Marseille, France

[3] AP-HM, Hôpital de Sainte Marguerite, Service de Rhumatologie, Pôle Appareil Locomoteur, 13005, Marseille, France.

[4] AP-HM, Hôpital de la Timone, CEMEREM, Pôle Imagerie Médicale, 13005, Marseille, France.

[5] Schulthess Clinic, Human Performance Lab, 8008 Zurich, Switzerland.

[6] University of Lausanne, Faculty of Biology and Medicine, Institute of Sport Sciences and Department of Physiology, Lausanne, Switzerland.

[7] Current affiliation : Institut NeuroMyoGène, Université Claude Bernard Lyon 1, INSERM U1217, CNRS UMR 5310, Villeurbanne, France.

* julien.gondin@univ-lyon1.fr

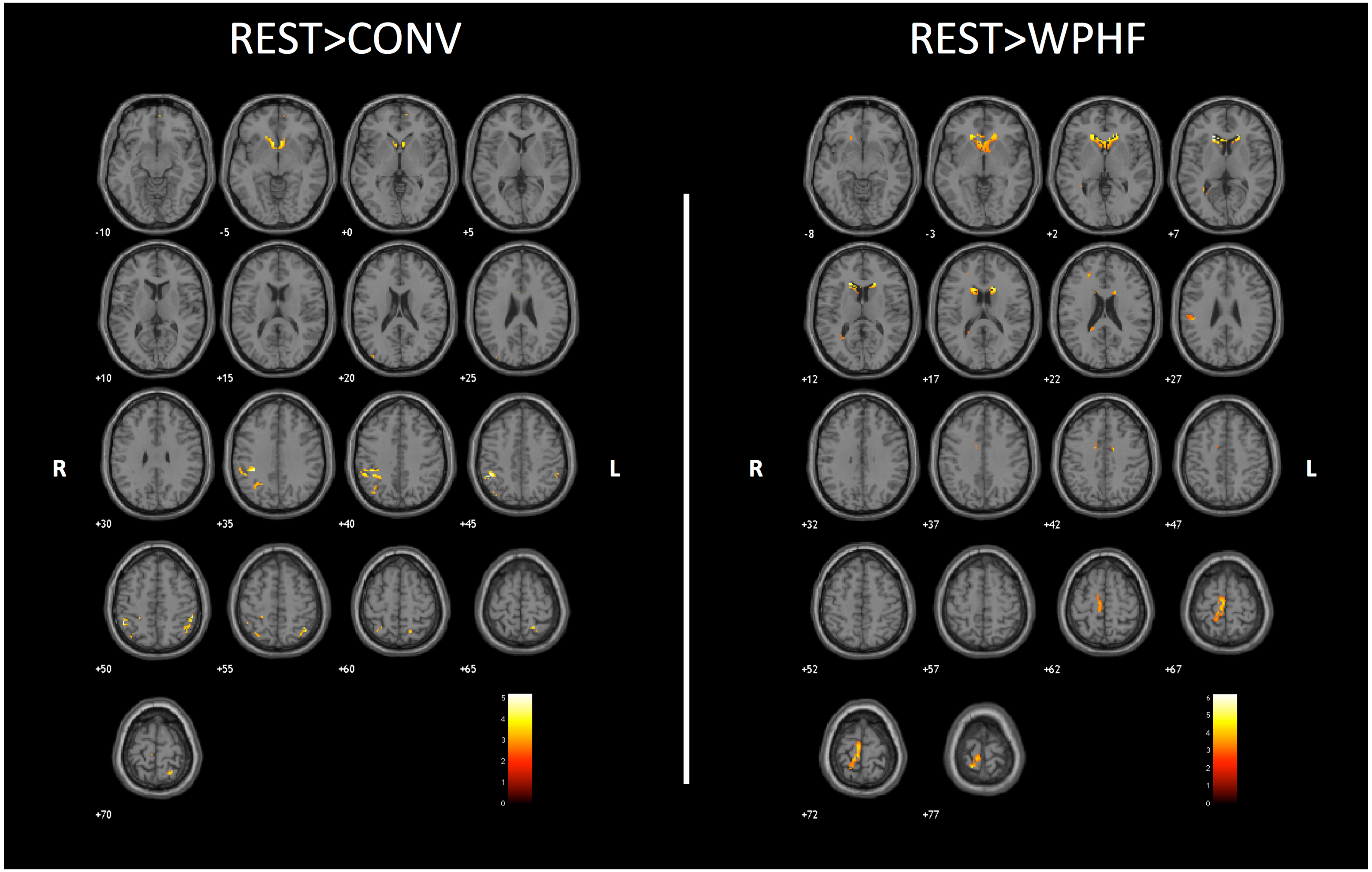

Supplemental Figure 1. Group deactivation maps ($p < 0.005$; k = 20; FDR corrected at cluster level $p < 0.05$) during CONV and WPHF protocols performed at 10% of maximal voluntary contraction force as compared to rest. Statistical maps were coregistered with the SPM-MNI single subject T_1 images. The color scale represents the T values. R: right hemisphere; L: left hemisphere.

SUPPLEMENTAL TABLE 1 - Brain regions activated during VOL, CONV and WPHF protocols compared to rest ***when considering the respective mean force as a regressor*** ($p < 0.005$; k = 20; FDR corrected at cluster level $p < 0.05$)

Protocols	Regions (Brodmann area)	Side	Cluster size (k)	MNI coordinates			T
				x	y	z	
VOL>REST	*Posterior cerebellum*	R	28835	38	-68	-18	7.98
	Precentral gyrus (4)	L		-34	-18	54	7.75
	Posterior cerebellum	R		46	-60	-22	7.73
	Middle occipital gyrus (19)	R		26	-84	14	7.09
	Fusiform gyrus (37)	L		-50	-64	-14	6.83
	Fusiform gyrus (19)	L		-24	-64	-6	6.62
	Orbital gyri (18)	L		-38	-84	4	6.59
	Anterior cerebellum	L		-6	-46	0	6.47
	Putamen	R		32	-10	-8	6.46
	Cuneus (18)	L		-22	-94	14	6.38
	Posterior cerebellum	R		26	-78	-14	6.28
	Precuneus (7)	L		-4	-56	50	6.27
	Lingual gyrus (17)	R		18	-90	-4	6.27
	Lingual gyrus (18)	L		-22	-90	4	6.24
	Middle occipital gyrus (19)	R		34	-74	0	6.23
	Putamen	L		-32	-16	-6	6.2
CONV>REST	*Lingual gyrus (19)*	L	6374	-28	-68	-8	6.53
	Anterior cerebellum	L		-2	-42	-24	6.51
	Posterior cerebellum	R		22	-84	-16	6.48
	Anterior cerebellum	R		12	-56	-16	6.13
	Anterior cerebellum	L		-18	-58	-18	6.13
	Lingual gyrus (18)	R		6	-68	2	5.82
	Posterior cerebellum	L		-18	-72	-16	5.82
	Anterior cerebellum	R		10	-48	-6	5.77
	Cerebellum	R		4	-44	4	5.64
	Posterior cerebellum	R		22	-66	-18	5.62
	Anterior cerebellum	R		20	-62	-20	5.6
	Posterior cerebellum	R		28	-68	-22	5.42
	Posterior cerebellum	R		12	-72	-16	5.38
	Posterior cerebellum	L		-18	-66	-16	5.37
	Orbital gyri (18)	L		-38	-84	4	5.31
	Lingual gyrus (18)	L		-24	-56	0	5.15

(continued on next page)

Protocols	Regions (Brodmann area)	Side	Cluster size (k)	MNI coordinates			T
				x	y	z	
CONV>REST	*Insula*	L	2126	-34	-20	10	6.21
	Inferior parietal lobule (40)	L		-64	-16	12	5.94
	Putamen	L		-30	-4	-10	5.46
	Superior temporal gyrus (22)	L		-54	-10	-10	5.44
	Superior temporal gyrus (22)	L		-56	-16	-8	5.12
	Superior temporal gyrus (22)	L		-52	-16	-4	5.03
	Superior temporal gyrus (38)	L		-46	8	-24	4.98
	Hippocampus	L		-32	-8	-20	4.83
	Caudate nucleus	L		-36	-22	-8	4.73
	Insula	L		-44	-6	-6	4.47
	Hippocampus	L		-28	-28	-8	4.37
	Putamen	L		-30	14	-6	4.36
	Precentral gyrus (6)	L		-48	-4	4	4.24
	Putamen	L		-28	8	-6	4.19
	Inferior parietal lobule (40)	L		-54	-22	14	3.88
	Caudate nucleus	L		-34	-30	-2	3.87
	Caudate nucleus	R	776	14	6	-14	6.15
	Thalamus	R		0	-6	0	5.92
	Thalamus	L		-4	-2	4	5.83
	Thalamus	R		8	-2	-8	5.07
	Caudate nucleus	L		-18	-4	18	3.44
WPHF>REST	*Precentral Gyrus (4)*	L	4286	-60	-4	20	5.96
	Fusiform gyrus (19)	L		-32	-64	-10	5.14
	Fusiform gyrus (19)	L		-28	-66	-8	4.77
	Posterior cerebellum	R		32	-70	-10	4.69
	Putamen	L		-24	-18	8	4.68
	Parahippocampal gyrus (19)	L		-26	-50	-6	4.67
	Lingual gyrus (18)	L		-28	-40	-16	4.66
	Anterior cerebellum	R		16	-78	-6	4.57
	Fusiform gyrus (37)	L		-24	-44	-14	4.57
	Lateral globus pallidus	L		-28	-20	0	4.38
	Parahippocampal gyrus (36)	L		-30	-36	-14	4.37
	Middle temporal gyrus (22)	L		-46	-30	4	4.31
	Anterior cerebellum	L		-32	-52	-12	4.17
	Lingual gyrus (18)	R		10	-86	8	4.15
	Fusiform gyrus (37)	R		30	-42	-16	4.15
	Lingual gyrus (18)	R		10	-86	0	4.14

SUPPLEMENTAL TABLE 2 – Differences in brain activation between VOL and NMES protocols

($p < 0.005$; k = 20; FDR corrected at cluster level $p < 0.05$)

Contrasts	Regions (Brodmann area)	Side	Cluster size (k)	MNI coordinates			T
				x	y	z	
VOL>CONV	*Cuneus (17)*	R	14357	12	-92	4	7.57
	Lingual gyrus (18)	R		18	-90	0	7.41
	Middle temporal gyrus (18)	R		24	-88	8	6.7
	Middle temporal gyrus (19)	R		24	-82	18	6.58
	Middle temporal gyrus (18)	L		-22	-92	12	6.11
	Inferior temporal gyrus (19)	R		34	-82	4	6.03
	Precuneus (7)	R		4	-70	56	5.72
	Cuneus (17)	L		-12	-90	14	5.67
	Middle occipital gyrus (18)	L		-18	-90	14	5.66
	Precuneus (7)	L		-8	-56	44	5.62
	Cuneus (17)	L		-14	-96	10	5.59
	Cuneus (17)	L		-10	-90	10	5.59
	Cuneus (17)	L		-18	-92	4	5.36
	Lingual gyrus (18)	L		-6	-82	6	5.16
	Lingual gyrus (18)	L		-12	-76	2	5.09
	Cuneus (19)	R		6	-86	36	4.99
VOL>WPHF	*Cuneus (17)*	L	12415	-14	-98	10	7.1
	Lingual gyrus (18)	R		18	-90	0	6.54
	Cerebellum	R		0	-86	-16	6.5
	Cuneus (17)	R		10	-92	6	6.36
	Middle temporal gyrus (18)	L		-22	-92	12	5.91
	Posterior cerebellum	R		22	-78	-14	5.83
	Inferior occipital gyrus (18)	R		32	-78	0	5.82
	Posterior cerebellum	L		-24	-72	-16	5.65
	Lingual gyrus (18)	L		-12	-86	-10	5.48
	Posterior cerebellum	L		-22	-64	-16	5.46
	Precuneus (7)	R		2	-70	56	5.34
	Anterior cerebellum	R		6	-70	-8	5.33
	Middle temporal gyrus (18)	R		22	-90	8	5.31
	Lingual gyrus (18)	L		-8	-92	-4	5.22
	Cuneus (19)	R		4	-86	38	5.18
	Anterior cerebellum	L		-2	-66	-4	5.16
CONV>VOL	*Caudate nucleus*	L	645	-10	6	14	5.86
	Thalamus	R		0	-6	2	5.47
	Caudate nucleus	R		6	-2	14	4.34
	Caudate nucleus	R		10	4	16	4.29
	Caudate nucleus	R		18	6	16	4.02
	Caudate nucleus	R		20	-2	14	3.4
	Putamen	R		26	4	10	3.05
	Putamen	R		26	0	8	2.81
	Putamen	R		24	-6	12	2.8
